# Advancing Toxicology Research Using *In Vivo* High Throughput Toxicology with Small Fish Models

**DOI:** 10.14573/altex.1601281

**Published:** 2016-06-21

**Authors:** Antonio Planchart, Carolyn J. Mattingly, David Allen, Patricia Ceger, Warren Casey, David Hinton, Jyotshna Kanungo, Seth W. Kullman, Tamara Tal, Maria Bondesson, Shawn M. Burgess, Con Sullivan, Carol Kim, Mamta Behl, Stephanie Padilla, David M. Reif, Robert L. Tanguay, Jon Hamm

**Affiliations:** 1 Department of Biological Sciences, North Carolina State University, Raleigh, NC, USA; 2 Center for Human Health and the Environment, North Carolina State University, Raleigh, NC, USA; 3 Integrated Laboratory Systems, Inc., Research Triangle Park, NC, USA; 4 National Toxicology Program Interagency Center for the Evaluation of Alternative Toxicological Methods, National Institute of Environmental Health Sciences, Research Triangle Park, NC, USA; 5 Nicholas School of the Environment, Duke University, Durham, NC, USA; 6 National Center for Toxicological Research, U.S. Food and Drug Administration, Jefferson, AR, USA; 7 Integrated Systems Toxicology Division, National Health and Environmental Effects Research Laboratory, Office of Research and Development, U.S. Environmental Protection Agency, Research Triangle Park, NC, USA; 8 Department of Pharmacological and Pharmaceutical Sciences, University of Houston, Houston, TX, USA; 9 National Human Genome Research Institute, Bethesda, MD, USA; 10 Department of Molecular & Biomedical Sciences, University of Maine, Orono, ME, USA; 11 Graduate School of Biomedical Science and Engineering, University of Maine, Orono, ME, USA; 12 Division of National Toxicology Program, National Institute of Environmental Health Sciences, Research Triangle Park, NC, USA; 13 Department of Environmental & Molecular Toxicology, Oregon State University, Corvallis, OR, USA

**Keywords:** aquatic models, 21^st^ century toxicology, alternatives

## Abstract

Small freshwater fish models, especially zebrafish, offer advantages over traditional rodent models, including low maintenance and husbandry costs, high fecundity, genetic diversity, physiology similar to that of traditional biomedical models, and reduced animal welfare concerns. The Collaborative Workshop on Aquatic Models and 21^st^ Century Toxicology was held at North Carolina State University on May 5-6, 2014, in Raleigh, North Carolina, USA. Participants discussed the ways in which small fish are being used as models to screen toxicants and understand mechanisms of toxicity. Workshop participants agreed that the lack of standardized protocols is an impediment to broader acceptance of these models, whereas development of standardized protocols, validation, and subsequent regulatory acceptance would facilitate greater usage. Given the advantages and increasing application of small fish models, there was widespread interest in follow-up workshops to review and discuss developments in their use. In this article, we summarize the recommendations formulated by workshop participants to enhance the utility of small fish species in toxicology studies, as well as many of the advances in the field of toxicology that resulted from using small fish species, including advances in developmental toxicology, cardiovascular toxicology, neurotoxicology, and immunotoxicology. We also review many emerging issues that will benefit from using small fish species, especially zebrafish, and new technologies that will enable using these organisms to yield results unprecedented in their information content to better understand how toxicants affect development and health.

## 1 Introduction

Traditional methods for testing the biological activity and toxicity of chemicals are time-consuming and costly. As a result, only a fraction of chemicals in commerce have been fully characterized for their potential hazard and risks to human health and the environment. While high-throughput *in vitro* cell-based assays have the potential to more efficiently provide insight into the mechanisms of action associated with the tens of thousands of chemicals lacking adequate toxicity data (Attene-Ramos et al., 2013a,b; Huang et al., 2011, 2014; Sun et al., 2012a; Yamamoto et al., 2011), these assays do not fully recapitulate the developmental, physiological, and disease processes observed in the whole animal.

The use in toxicity testing of small fish models including *Danio rerio* (zebrafish) can potentially address these limitations. In addition to low maintenance and husbandry costs, high fecundity, and genetic diversity, fish models have the added benefit of reduced animal welfare concerns, particularly during embryonic stages. The National Institutes of Health Office of Laboratory Animal Welfare (NIH OLAW) considers aquatic models “live, vertebrate animals at hatching” and approximates zebrafish hatching at 72 hours post fertilization^1^. Thus, NIH OLAW does not require inclusion of pre-hatching zebrafish embryos in the Animal Requirements section of an Animal Study Proposal. Furthermore, NIH OLAW states that zebrafish larvae younger than 8 days post-fertilization are incapable of feeling pain or distress, supporting their use in longer term studies without incurring significant animal welfare concerns.

Despite their many advantages (Bugel et al., 2014), fish models remain relatively modest contributors to the field of toxicology. To highlight and consider the key role small fish and fish embryos may play in toxicology research and testing, the National Toxicology Program, North Carolina State University, and the U.S. Environmental Protection Agency convened an international Collaborative Workshop on Aquatic Vertebrate Models and 21^st^ Century Toxicology on May 5-6, 2014, at North Carolina State University in Raleigh, North Carolina.

The goals of the workshop were to explore and discuss how aquatic models, and in particular small fish models, may be used to (1) screen and prioritize compounds for further *in vivo* testing and (2) assess mechanisms of chemical toxicity. The workshop had five specific objectives:

– To encourage interactions between *in vitro* toxicologists and biomedical scientists using fish models, thus facilitating the translation of experimental approaches in these models into novel toxicity tests, adverse outcome pathway assessments, and mode-of-action discovery
– To raise awareness within the toxicology field of the advantages of fish models, including availability of genetic and genomic information; transgenic resources; molecular tools; low cost and ease of maintenance; rapid, external embryonic development; and ability to perform high-throughput studies in a vertebrate animal
– To develop a framework for integrating toxicology data derived from fish models with ongoing *in silico*, *in vitro*, and *in vivo* testing initiatives to enhance risk and safety assessments of chemicals and pharmaceuticals
– To explore the potential for fish models to aid in identifying genetic contributions to human exposure susceptibility and to anchor phenotypic outcomes of exposure to mechanisms of action
– To identify and prioritize future research initiatives using fish models to address current information gaps, including improvements in risk and safety assessments for multiorgan toxicity, longitudinal studies to assess long-term consequences of chronic exposures, the embryonic basis of adult disease, and multi-generational effects of exposure to environmental contaminants

Experiments using fish models, particularly zebrafish but also other small fishes including medaka (*Oryzias latipes*) and killifish (*Fundulus heteroclitus*), are driving important contributions to our understanding of toxicity in the cardiovascular, immune, developmental, behavioral, and nervous systems. These models are being used in high-throughput methods and approaches that are transforming toxicology. This review summarizes the proceedings of the workshop, which centered on emerging issues of importance to toxicology and overviews of the role of small fish models in developmental, cardiovascular, nervous system, and immune system toxicology, as well as the development of new technologies that will facilitate the increased use of small fish models in toxicology research, especially in future large-scale chemical screens.

## 2 Emerging issues

Human health is impacted by lifetime exposure to stressors, including pathogens and chemicals in the air, food, and water. The mission of the National Institute of Environmental Health Sciences is to “discover how the environment affects people in order to promote healthier lives”^2^ . In its 2012-2017 Strategic Plan, the institute states as a strategic goal identifying and understanding “fundamental shared mechanisms or common biological pathways… in order to enable the development of applicable prevention and intervention strategies.” The focus on *“shared mechanisms*” (i.e., evolutionary conservation) requires animal model systems with properties, both biological and technological, suitable to the task. In this section we review how the properties of small fish models, including their similarities to humans, can be leveraged to support a better understanding of gene-environment interactions that affect human health.

### 2.1 Screening large sets of chemicals

Individuals are exposed to thousands of complex chemical mixtures in the environment. Exposure can vary by age, geography, occupation, and socioeconomic status. Determining how chemicals in these mixtures might interact to adversely affect health is exceedingly difficult. Only a small proportion of the many thousands of chemicals manufactured and used worldwide have been thoroughly tested for potential toxicity. Furthermore, the U.S. Toxic Substances Control Act (TCSA) (United States Congress, 1976) exempted over 60,000 chemicals already in use at the time the act was adopted from toxicity tests. Thus, the small number of chemicals that require thorough review for toxicity are those classified as “new chemicals”, i.e., not present on the TCSA inventory. Toxicity analysis is further hampered by the prohibitive expense and time required for testing in mammalian systems (National Research Council, 2007). In contrast, the European REACH (Registration, Evaluation, Authorization and Restriction of Chemicals) regulation requires companies to register all manufactured substances, and enforcement of REACH provides mechanisms for banning substances if hazard risks linked to their use cannot be adequately managed (Barr, 2007). The National Academy of Sciences, recognizing the shortcomings of traditional approaches to address complex issues, challenged the toxicology field to rapidly develop relevant toxicological data to guide predictions of biological responses in human tissues (Gibb, 2008). Significant federal and commercial resources were invested to develop high-throughput cell culture assays to assess the toxicity of thousands of chemicals and mixtures. While these approaches have generated volumes of data on many chemicals for which limited hazard information was available, they lack the ability to recapitulate the physiological processes in an intact organism. Fish models are gaining widespread acceptance as an alternative to *in vitro*, cell-based models in which to study the effects of complex chemical mixtures on whole-animal phenotypes.

Vertebrates are most susceptible to environmental insult during early development, a time during which most signal transduction pathways are active (Pauli et al., 2012). The use of an *in vivo* model such as zebrafish embryos in high-throughput screening efforts increases the probability of identifying adverse interactions between a chemical or mixture and biological targets. Fish embryos have been used to identify molecular mechanisms altered by exposure to broad chemical classes, including pesticides, herbicides, recreational drugs, dioxins, PCBs, flame retardants, fluorinated compounds, polycyclic aromatic hydrocarbons, nanoparticles, and metals (Guo, 2009; Hans et al., 2013; Hermann and Kim, 2005; Garcia-Reyero et al., 2014; Wang et al., 2016). Importantly, these studies have helped identify the pathways involved in chemical-specific adverse outcomes (Yozzo et al., 2013b).

Major advances in instrumentation and assay development have resulted in robust platforms for high-throughput *in vivo* chemical testing in zebrafish. These advances include automated embryo manipulation, handling, and imaging of early life stages yielding unprecedented rates of phenotype discovery (Graf et al., 2011; Letamendia et al., 2012; Mandrell et al., 2012; Pardo-Martin et al., 2010; Vogt et al., 2009). These approaches exploit the small size, transparency, and fecundity of zebrafish for automated and high-throughput morphometric and behavioral phenotyping (Bugel et al., 2014; Pardo-Martin et al., 2013; Petzold et al., 2010; Rihel et al., 2010; Truong et al., 2014). For example, emerging data indicate that the zebrafish is a powerful model for studying the causes of human brain disorders, including mechanisms by which environmental compounds may perturb neurodevelopment or contribute to Parkinson’s disease and other later-life neurological disorders (Kalueff et al., 2014). Screening of potential neurotoxicants has been assisted greatly by development of instruments in which simultaneous behavioral measurements (e.g., distance moved, swimming speed) can be made in a 96-well dish containing one larva per well (Padilla et al., 2012; Truong et al., 2014; Xi et al., 2011).

Recent efforts have demonstrated that small fish species can be important elements of high-throughput testing programs to test environmental chemicals and mixtures. Proof-of-concept studies on the U.S. Environmental Protection Agency’s ToxCast Phase I chemicals compared zebrafish data to available *in vitro* and *in vivo* toxicity data. The results support the hypothesis that biological responses to chemical exposures are evolutionarily conserved and reflect the existence of common biological pathways shared across phyla as targets of exposure (Raftery et al., 2014; Sipes et al., 2011; Truong et al., 2014; Yozzo et al., 2013a). Similarly, the U.S. federal interagency Tox21 program (Bucher, 2013; Mahadevan et al., 2011; Tice et al., 2013) uses a variety of *in vitro* and *in vivo* assays, including zebrafish embryos, to screen environmental chemicals for potential hazards. The insight gained into the structural basis for chemical bioactivity will facilitate predictive modeling and provide data to support risk assessments, in turn supporting “green chemistry” goals of developing high-performing chemical products that are safer for the environment.

### 2.2 Rapid mechanistic evaluations

An additional strength of fish embryo models is the availability of genetic and molecular tools to rapidly explore toxicity mechanisms. These include unbiased “omics” approaches in the whole animal, which provide a powerful way to identify pathways perturbed by environmental exposures (Groh and Suter, 2015; Scholz et al., 2008). Transcriptomics, proteomics, epigenomics, and metabolomics can facilitate the translation of zebrafish data across species (Williams et al., 2014). These global approaches can then help to identify candidate genes for hypothesis testing. Genome-wide evaluations provide a comprehensive view of molecular integration and will assist in identifying conserved pathways that underlie chemical toxicity. Transient and stable genetic approaches now routine in zebrafish (discussed below) have produced large numbers of custom zebrafish lines that can be used to define the roles of specific gene targets in chemical toxicity, enabling researchers to efficiently proceed from candidate identification to *in vivo* hypothesis testing. Other small fish, including medaka, can be made isogenic, which could facilitate toxicology studies that require baseline phenotypic uniformity, such as the effect of exposures on epigenetic modifications that alter metabolic pathways.

### 2.3 Essential needs to maximize impact of aquatic models

Despite the many advantages inherent in using fish models for environmental health research, the existence of detailed protocols from the U.S. EPA^3^ and the Organization for Economic Co-operation and Development^4^ (OECD) for using zebrafish embryos in toxicity assays, and established husbandry protocols available from the Zebrafish International Resource Center^5^ (ZIRC), obstacles to the broad use of these models for hazard identification and mechanistic evaluations persist and include (1) inadequate description of strains, (2) inconsistent husbandry practices, including use of undefined and variable diets, (3) inadequate disease surveillance, and (4) lack of standardization and validation of toxicity assays.

Decades of mammalian toxicology research have demonstrated that choice of strain variation greatly influences chemical susceptibility (Bradford et al., 2011). Therefore, accurate reporting of strains used for chemical testing and phenotype discovery is critically important. Similarly important is the accurate documentation of diet. It is well established that diet, as the most significant environmental exposure, influences molecular signaling (O’Prey et al., 2003). Recent studies in zebrafish revealed that parental diet influences the basal embryonic transcriptome (Miller et al., 2014); therefore, poorly documented diets risk clouding data interpretation across laboratories. Additionally, fish, like other vertebrate models, are susceptible to pathogens that threaten colony health and egg production, and uncontrolled infections have the potential to influence research results. Standard practices for strain documentation, husbandry, and disease surveillance would facilitate comparison of experimental results across laboratories and support broader acceptance of fish models.

A number of experimental design considerations can influence variability and thereby interpretation and translation in early life-stage hazard identification assays using fish models. Chemical purity is particularly important: Since developing zebrafish can be exquisitely sensitive to chemical exposures, a highly potent contaminant present at very low concentrations within the test chemical preparation could be a source of false positives. Organic solvents (such as ethanol, dimethyl sulfoxide and acetone), which are often used to increase water solubility of test chemicals, can potentially influence chemical response endpoints. The response to a chemical might also be influenced by the exposure regimen; for example, some screening protocols use static exposures during embryonic development, while others specify renewal of media every 24 hours. The composition of the testing plates (glass, plastic) can also influence chemical uptake and response, as can the number of animals in an individual well. Any of these parameters, in isolation or together, could influence whether a chemical is considered a “hit” in a screen. Moving forward, these variables must be minimized across laboratories to avoid data conflicts.

## 3 Developmental toxicology

### 3.1 Zebrafish and development

One of the most pressing problems in environmental health and toxicology is determining the role of gestational exposure to toxicants in the etiology of birth defects and adult-onset diseases. Instances of acute high-level exposure to toxicants during critical windows of development, such as organogenesis or nervous system development, are known to cause adverse effects resulting in organ failure and death. Such exposures may account for up to 10% of all severe birth defects (reviewed by Grajewski et al., 2005). More pernicious, however, are the effects of chronic low-dose gestational exposures from contaminated environments, medications taken during pregnancy, or parental lifestyle choices. The primary route of exposure is through the placenta, although a significant fraction of the exposure burden takes place through amniotic fluid (an aquatic environment) (Bradman et al., 2003; Graça et al., 2008), where in early stages of development absorption is likely to be transdermal working by mechanisms not dissimilar to those through which toxicants enter developing fish embryos. Toxicant exposures could affect development in ways that do not lead to obvious defects but instead induce long-term physiological changes potentiating later disease development, for example, diethylstilbestrol (Herbst et al., 1971, 1999). Potential mechanisms could include: altering cell-specific epigenetic profiles; creating long-lived protein adducts with altered functionality; altering the numbers of specific cell types, such as adipocytes; or subtly rewiring neuronal connections in the brain, with profound behavioral consequences. Complicating investigations into these effects is the fact that not all exposed individuals will be affected. Genetic variations or ameliorations in the embryonic environment (e.g., mother’s diet) may provide sufficient buffering to prevent cellular changes that otherwise lead to disease. Such buffering, which can be difficult to characterize, can increase the threshold dose needed to cause effects leading to disease.

Zebrafish have been used to investigate the effects of chronic low-dose gestational exposures on physiology and adult-onset diseases. Zebrafish have higher fecundity than rodents; their bodies, which are transparent during organogenesis, allow easy assessment of early developmental effects; and their organ systems are similar to mammals. Zebrafish studies also allow for control of confounding variables that could affect risk assessment, which is not possible in epidemiological studies.

In the last two decades studies using fish models have moved beyond traditional developmental biology to include studies measuring the effects of chronic, low-dose environmental exposures on the development of disease (reviewed by Bugel et al., 2014). This new direction came at a time when such exposures to environmental toxicants, including food additives, pollutants, and metals, were suspected in a number of human disorders rooted in development, such as limb defects, cardiovascular abnormalities, childhood obesity, immune disorders, neurological and behavioral disorders, and cancer. Exposure studies that use fish models to identify environmental factors involved in the development of these complex, polygenic diseases have yielded interesting results and may represent a way to unravel the complex interplay between environment, development and disease (Ito et al., 2010; Mattingly et al., 2009).

### 3.2 Genome duplications in fish as a source of insight into developmental toxicity

The possibility that a disorder may arise from environmental exposures during development can be affected by allelic variations in a number of genes, both in humans and fish. One advantage of fish models over other model organisms for investigating these types of effects is the presence of a fish-specific whole genome duplication (Taylor et al., 2003; Taylor and Raes, 2004). Individual duplicate genes, known as paralogs, are in some cases functionally retained with modifications. These include the emergence of new functions, termed neofunctionalization, or the partition of the ancestral function among newly duplicated genes, termed subfunctionalization (reviewed by Innan and Kondrashov, 2010). The subfunctionalization phenomenon has provided insight into differential susceptibilities of a population to diverse chemical exposures, including how allelic differences can influence resistance to specific compounds (Reitzel et al., 2014). Gene duplications in bony fish have also yielded insights into poorly understood transcriptional regulatory mechanisms important in development, including the role of evolutionarily conserved transcription factors such as the aryl hydrocarbon receptor (AhR) and Nuclear Factor (Erythroid-Derived 2)-Like 2, for which fish have multiple paralogs (Garner et al., 2013; Karchner et al., 2005). Studies in zebrafish embryos indicate that environmentally dependent changes in developmental stage-dependent glutathione redox dynamics may alter spatial-temporal expression of a subset of these paralogs, thus pointing to one possible mechanism of developmental toxicity (Timme-Laragy et al., 2013). It is important, however, to remain vigilant of the possibility that functional compensation is afforded by having multiple paralogs of a gene. These and other studies in fish models will be invaluable in the effort to understand how chemical exposures during embryogenesis may lead to diseases in adulthood.

### 3.3 Endocrine disrupting compounds, development, and health

Endocrine-disrupting compounds are found throughout the environment and are suspected to cause several human metabolic and reproductive disorders (Diamanti-Kandarakis et al., 2009). The ability of many endocrine-disrupting compounds to partition in water has led to the use of fish models, including zebrafish, medaka, killifish, and mosquitofish (*Gambusia affinis*), in studies on their effects on health. These studies have shown endocrine-disrupting compounds can cause perturbations of developmentally critical pathways, including hedgehog signaling, fibroblast growth factor (FGF) signaling and Hox pathways (Lee et al., 2013; Hill et al., 2003; Schiller et al., 2013). While investigation of these perturbations is still at an early stage, it appears that disrupting these pathways may underlie several commonly observed endocrine disorders, including altered sex determination, feminization, and masculinization. They may also cause unexpected effects such as inhibition of regenerative potential, which could inhibit the ability of affected individuals to recover from injuries to the liver or other organs capable of regeneration.

### 3.4 Using fish models in genetic studies of human diseases

The Human Genome Project signaled a major paradigm shift across many areas of biomedical research, particularly in the areas of clinical human genetics and drug development. Recent technological advances in aquatic model research have had a profound impact on these two important areas.

GWAS Central^6^ is a public database of findings from human genome-wide association studies. It currently contains data from over 1,800 studies, representing many thousands of loci contributing to human diseases ranging from autism to tuberculosis susceptibility. Many of these diseases trace their origins to misregulated developmental programs (e.g., autism and obesity), wherein environment and environmental exposures are suspected causal agents (Rzhetsky et al., 2014). However, it remains to be demonstrated that any of these loci actually cause disease. One approach to confirming the causative effect of a candidate disease gene is to demonstrate similar phenotypes in animal models by gene inactivation, commonly known as a gene “knockout.” For decades, mouse knockouts have been used to validate the functions of vertebrate genes, but large-scale identification of candidate genes in mice is limited by many factors, including low fecundity, *in utero* lethality, and expensive husbandry. With the advent of genome editing via targeted zinc-finger nucleases (Doyon et al., 2008; Foley et al., 2009), transcription activator-like effector nucleases (TALENs) (Cade et al., 2012; Moore et al., 2012) and CRISPR-Cas (clustered regularly interspaced palindromic repeats) (Jao et al., 2013), it is now possible to validate candidate loci on a large scale in fish models.

A specific example of how fish models can be effectively used to confirm the cause of human genetic diseases comes from the National Institutes of Health’s Undiagnosed Diseases Network^7^. In this program, patients with difficult-to-diagnose diseases, including syndromic disorders resulting in adverse developmental outcomes, undergo a careful clinical workup, and genetic samples are obtained from them and direct family members (Gahl et al., 2012). The genetic samples are used for sequencing all exons encoded by the genome (the exome), which often generates many potential candidate genes. The program is now studying 5-10 patient-identified candidate genes in zebrafish using CRISPR-Cas at less than $100 per knockout. Using this approach many hundreds of genes can be rapidly tested. In addition, CRISPR-Cas editing of the fish genomes using short, single-stranded oligos as a template (Auer et al., 2014; Bedell et al., 2012; Zu et al., 2013) opens the door for possible approaches to model human diseases by genome editing of fish models with a mutation to demonstrate causative effects from sequence variants.

One challenge of genome-wide association studies is the fact that nearly half of the strong association signals land in intergenic regions, presumably because the variants are within regulatory regions. Testing the functionality of regulatory elements can be effectively accomplished in many aquatic models by linking putative enhancer sequences to minimal promoter sequences that drive expression of fluorescent reporters (Fisher et al., 2006; Ishibashi et al., 2013).

## 4 Emerging technologies

The growing importance to toxicology of aquatic models, and in particular zebrafish, is largely due to emerging technologies that position these models to address complex, system-wide questions about susceptibility and disease etiologies. These emerging technologies include genome editing, scalable low- and medium-throughput screening, whole-animal imaging, and associated computational approaches.

### 4.1 Genome editing

As stated previously, genetic approaches for generating mutant lines in zebrafish are invaluable for examining gene function and identifying disease models. The need for targeted induction of loss-of-function mutations stimulated significant innovations in zebrafish, including retroviral insertional mutagenesis, morpholinos, targeting induced local lesions in genomes (TILLING), Tol2 transposition, and genome editing via zinc finger nucleases (Cade et al., 2012; Huang et al., 2012). More recent developments of TALENs and the CRISPR-Cas system offer even greater advantages with respect to cost, time, efficiency, specificity and overall success in generating precise and heritable gene edits in many aquatic models but especially zebrafish (Hwang et al., 2013; Jao et al., 2013; Jinek et al., 2012). For toxicology studies, these techniques provide novel tools for answering questions about the effects of exposures on specific molecular pathways, susceptibility, and environmentally influenced disorders and birth defects.

### 4.2 Whole animal imaging

The ability to assess subtle effects at the cellular or tissue level is critical when evaluating gene function and the short- and long-term phenotypic consequences of exposure. Several innovative imaging options have emerged in recent years to address this need. For example, it is now possible to auto-load fish embryos from a 96-well plate into a capillary tube mounted on a confocal microscope. The tube can then be automatically oriented to visualize embryos at high resolution (Pardo-Martin et al., 2010). Additional tools including image recognition algorithms and optimal capillary materials have been developed in parallel to further increase throughput (Chang et al., 2012). Public online reference databases provide high-resolution and deep anatomical coverage of the developing and adult zebrafish (noted in Eames et al., 2013). In addition, micron-scale tomography devices enable three-dimensional reconstruction of a fixed specimen from hundreds or thousands of X-ray images taken at varying angles. The whole zebrafish can be imaged by micron-scale tomography, thereby providing opportunities to learn about the developing and adult vertebrate architecture. Synchrotron-based micron-scale tomography also presents innovative and high-throughput opportunities for anatomical phenotyping (Cheng et al., 2011, 2012). Many of these technologies have not been fully exploited and their relative advantages and potential contributions to toxicology are still being realized.

### 4.3 Computational analysis

High-throughput experiments generate immense datasets; the scale and diversity of the resulting data present new analytical challenges. For example, an experiment may include broad concentration-response spacing that brackets predicted toxicity ranges for many chemicals. Results of these experiments may then be integrated with those of targeted followup experiments using narrower dose-response spacing. In this scenario it may be tempting to apply different analytical approaches to the broad vs. narrow data sets; however, this approach introduces problems in interpretation and generalizability (Guo and Bowman, 2008). Rather, similar analytical approaches should be used and appropriately parameterized for both ends of the scale to maximize the complementarity of large scale and targeted results. Although the basic analytical framework should be consistent across data scales, the suitability of analysis methods will differ with respect to the experimental goals. This is especially true for high-dimensional data, where issues such as multiple testing and batch effects must be addressed. Several methods, including conditional regression, hierarchical Bayesian approaches, and multivariate machine-learning methods, may be used to account for these (sometimes nested) contexts (Wilson et al., 2014). Regardless of method, solid experimental designs that include repeated controls must be used to assess batch effects and align results across laboratories or related platforms, and analyses should be suited to the study goals and provide output that is statistically robust and fit-for-purpose (Reif et al., 2015).

Functionally, data integration for systems biology-toxicology studies will require heavy investment in software infrastructure. In other fields facing similar challenges, some standardization of both methods and reporting has paid major dividends; examples include the “Tuxedo Suite” and associated data formats for next-generation sequence analysis (Trapnell et al., 2012). For emerging aquatic data streams, software pipelines will be required for repeatable analysis and reliable combination of data sources. These pipelines must accommodate newly generated data and standardize outputs for integration with other data sources to maximize the capacity to understand mechanisms of action, predict toxicity, and improve risk assessment (Krewski et al., 2014).

## 5 Cardiovascular toxicology

### 5.1 Small fish as models for cardiovascular development

Small fish species have characteristics that make them particularly suitable for identifying cardiovascular disruptors, enabling fish models to contribute significantly to our understanding of cardiovascular development and associated molecular pathways. Although fish hearts are two-chambered, they have coronary vasculature, contraction rate, and QT intervals similar to humans. Cardiovascular developmental pathways are highly conserved between fish models and higher vertebrates (Asnani and Peterson, 2014; Wilkinson and van Eeden, 2014). The molecular mechanisms underlying vessel formation that are conserved between humans and zebrafish include vascular endothelial growth factors and receptors, bone morphogenetic proteins, notch signaling molecules, semaphoring-plexin guidance factors, chemokines and adhesion molecules such as cadherins (reviewed by Gore et al., 2012; Schuermann et al., 2014; Wilkinson and van Eeden, 2014). Several genetic components required for cardiac development and function are also highly conserved. For example, the ether-a-go-go-related potassium channel, which contributes to the process of action potential repolarization, has a 99% amino acid identity in key domains between humans and zebrafish (Langheinrich et al., 2003).

A number of methods have been developed for screening of cardiovascular-disrupting compounds in fish models. Although the zebrafish cardiovascular system is functional by 72 hours post-fertilization, the embryo can survive without a functioning cardiovascular system for several days, presumably because diffusion through the skin is sufficient for gas exchange in early life stages (Cha and Weinstein, 2007; Tal et al., 2014). During this window of opportunity, genetic or chemical cardiovascular disruption can be studied in viable embryos. These studies are facilitated by transgenic fish that express fluorescent proteins in specific cardiovascular structures (reviewed by Heideman et al., 2005 and Schuermann et al., 2014). Studies using these models reveal the dynamics of vascular development and enable exact measurement of morphological changes during both normal and perturbed angiogenic processes (Kaufmann et al., 2012; Shirinifard et al., 2013). Cardiotoxicity screening methods have been developed that allow measurement of the heart rate of wild-type or transgenic fish expressing green fluorescent protein exclusively in myocardium and collection of electrocardiography data *in vivo* in embryos, larvae, and adult fish (Dhillon et al., 2013; Wen et al., 2012 and references therein). Methods also exist for rapid isolation of adult zebrafish hearts for *ex vivo* functional toxicity assays measuring atrial and ventricular pulsations (Kitambi et al., 2012; Lai et al., 2014). *In vivo* heart voltage dynamics can be visualized in the *cmlc2:mermaid* transgenic fish (Tsutsui et al., 2010).

### 5.2 Fish models in high-throughput screening studies to identify cardiovascular toxicants

McCollum et al. (2011) have summarized the use of fish models to identify environmental toxicants that perturb cardiovascular development and function. Surprisingly, only a few studies have utilized fish models in primary high-throughput assays for vascular toxicity screening. One example is described by Kitambi and colleagues (2009), who used zebrafish to screen a library of over 2,000 small molecules selected to provide a wide range of biological activities and structural diversity. This screen identified five vascular disruptors, four of which affected retinal vessel morphology at concentrations that did not cause apparent changes in the trunk vasculature. Tran et al. (2007) screened the LOPAC1280 compound library of pharmacologically active compounds and identified two known and one novel vascular disruptor. Despite these successes, zebrafish have mostly been used as an *in vivo* model to confirm *in vitro* screening results or to assess a limited number of molecules of interest. For example, combined targeted/phenotypic screening approaches have been used to identify anti-angiogenic agents as new compounds for cancer therapy (Chen et al., 2012; Evensen et al., 2013; Lee et al., 2013; Radi et al., 2012).

Fish models have mostly been used to screen chemicals for cardiotoxicity in drug discovery contexts. Studies using fish models to examine cardiotoxicity of environmental contaminants include a study examining the cardiotoxic effects of different types of crude oil in marine medaka (*Oryzias melastigma*; Zhang and Yan, 2014) and inclusion of endpoints such as pericardial edema in large-scale screens of environmental pollutants in zebrafish (Truong et al., 2014). Milan and colleagues (2003) screened 100 small molecules for effects on zebrafish embryo heart rates and identified a correlation between drugs causing bradycardia in zebrafish and those causing both QT prolongation and torsade de pointes (polymorphous ventricular tachycardia with marked QT prolongation) in humans. Despite these promising results, no high-throughput screening studies focusing solely on cardiotoxicity have been performed to date on libraries of environmental compounds.

The capacity of screening studies using zebrafish has been significantly improved by recent advances in automated arraying of embryos and chemicals and in imaging and image analysis, as described above. Automated high-content screening assays and vascular image analysis algorithms have been developed for measuring chemical effects on development of blood vessel structures, heart rate, and blood flow (Hans et al., 2013; Spomer et al., 2012; Yozzo et al., 2013a).

Small fish models are very useful for deciphering mechanisms and molecular initiating events of toxic compounds, including compounds that disrupt vasculogenesis and angiogenesis as described above. For human risk assessment, a three-tiered approach has been proposed that would use a combination of *in vitro*, *in vivo*, and systems biology-generated data to provide mechanistic information on toxic effects (Cote et al., 2012). However, to meet current throughput demands for toxicity testing, high-throughput screening methods that inform mechanisms of action will be required. A study using such methods was published by Kleinstreuer et al. (2011), who identified putative vascular disruptors by combining outputs from data mining analyses focused on genes affiliated with disruption of vascular development with high-throughput screening data for 309 compounds (ToxCast phase I library) tested using 467 mechanistic *in vitro* assays. From these data, an adverse outcome pathway for embryonic vascular disruption was proposed (Knudsen and Kleinstreuer, 2011). In follow-up experiments conducted in zebrafish, embryos were used to visualize and quantify blood vessel formation during development in response to a subset of putative vascular disruptors identified through *in vitro* screening (Tal et al., 2014). Findings in the zebrafish embryo were well correlated with *in vitro* signatures.

### 5.3 Opportunities to incorporate other technologies to detect cardiovascular toxicants

To complement high-throughput screening efforts, toxicogenomics tools should be applied to identify new molecular initiating events for cardiovascular disruption, keeping in mind that it is not unusual for one compound to affect several different apical endpoints (Lam et al., 2011). Relationships between gene products or potential biomarkers and apical effects could be clarified by performing overexpression or knockdown experiments using fish models, such as zebrafish and medaka, to validate the gene/protein-environment interaction in predicted pathways. In addition, to identify molecular events related to cardiovascular toxicity, tissue-specific “omics” analysis could be performed by dissecting tissues or sorting fluorescent cells from transgenic animals prior to transcriptomic or proteomic analyses. These techniques have been successfully implemented in studies of developmental changes in neuronal and gastrointestinal cells in zebrafish embryos (Cerda et al., 2009; Manoli and Driever, 2012; Stuckenholz et al., 2009) and could be readily adapted to cardiotoxicity studies. Such testing strategies are likely to increase both the number of identified cardiovascular toxicants and the understanding of their modes of action.

## 6 Neurotoxicology

Many seminal discoveries in neuroscience were made using aquatic models. For example, theories of neuronal cell membrane function and action potential were solidified by experiments that used the squid giant axon (Hodgkin et al., 1952; Young, 1938). Similarly, the sea slug provided the model for delineating the biological underpinnings of synaptic modifications during learned behaviors (Castellucci et al., 1978), and studies using goldfish were instrumental to understanding the biological basis of memory storage (Agranoff et al., 1965). While most recent neuroscience research has been conducted in rodents, aquatic vertebrates are enjoying renewed popularity for both basic neuroscience studies and neurotoxicology investigations.

### 6.1 Screening for nervous system perturbations

Although it is accepted that the nervous system is especially vulnerable to injury from a variety of chemicals, our understanding of the risks and mechanisms of injury is limited. To better assess the potential of untested chemicals to affect the nervous system, it is imperative that we develop faster and cheaper assays for neurotoxicity. Because zebrafish can be used in high-throughput applications, they are increasingly replacing or acting as adjuncts for traditional laboratory mammals in studies designed to screen for potential neurotoxins, determine the mechanism of neurotoxicity, or identify chemical effects on neural pathways (examples include: de Esch et al., 2012; Green et al., 2012; Kalueff et al., 2014; Kinth et al., 2013; Padilla and MacPhail, 2011; Parker et al., 2013; Peterson et al., 2008).

The fundamentals of nervous system development in zebrafish are fairly well understood (reviewed by Blader and Strahle, 2000; Guo, 2009 and Young et al., 2011). Techniques have been developed for rapid evaluation of the effects of chemical exposures on the nervous systems of fish models, and we are beginning to understand how results of these studies may be used to predict human effects. As with studies using mammals, studies of neurotoxicity in fish models involve evaluation of sensory function, motor function, or aspects of cognitive function (Roberts et al., 2013) using morphological, biochemical, or behavioral endpoints. Behavioral endpoints integrate nervous system function and are thus particularly appropriate apical endpoints for screening. Over 150 separate behaviors of larval and adult zebrafish can be measured (reviewed by Kalueff et al., 2013); changes in these behaviors have been used to identify neurotoxic chemicals.

In general, screens focus on either an endpoint of concern or a mechanism of toxicity. Libraries of environmental chemicals and pharmaceuticals have been screened in zebrafish for various aspects of neurotoxicity (Berghmans et al., 2008; Milan et al., 2003; Ou et al., 2009; Richards et al., 2008; Selderslaghs et al., 2013; Sun et al., 2012b; Winter et al., 2008; Yang et al., 2009), either using chemical training sets or large numbers of chemicals with unknown effects. One study screened a 3968-compound library for neuroactivity in young zebrafish by assessing the rest/wake locomotor states in 4-7 day old larval zebrafish (Rihel et al., 2010). This study identified over 550 compounds that altered locomotor activity and/or the sleep/wake cycle. Using hierarchical clustering, the authors found that larval zebrafish behavior accurately cataloged the neuroactive compounds according to their mechanism of action in humans, leading to the astounding conclusion that human bioactive drugs can be classified using a simple behavioral assessment in zebrafish larvae. In a related study from the same laboratory (Kokel et al., 2010, 2013), younger zebrafish embryos (30 hours post-fertilization) were used to classify known human neuroactive drugs from a library of 13,976 chemicals using a rapid screen (photomotor response), which enabled clustering of novel molecules with known neuroactive potentials. As an example, one of the novel molecules clustering with the monoamine oxidase inhibitors was found to be a potent monoamine oxidase inhibitor when tested *in vitro*, thereby supporting the concept that a simple zebrafish behavioral assay can identify chemicals with neuroactive properties.

### 6.2 Recommendations and new discoveries

As the number and use of behavioral screens in small fish models, and in particular zebrafish, becomes widespread, laboratories conducting these studies must adopt consistent protocol designs that contribute to intra- and inter-laboratory reproducibility. An ongoing review of many past studies reveals no consistent protocol for study design, with variations noted in exposure duration, individual or group dosing, frequency of dosing (daily, weekly, etc.), developmental phase of exposure (i.e., “window” of exposure), time elapsed between exposure and assessment, and statistical methods of analysis. At a minimum, all protocols should indicate:

– The interval between dosing and testing
– Whether overt toxicity was present
– The time of peak effect when comparing drug potencies
– Whether assessments were blinded
– The study design and statistical methods used
– How the raw data can be obtained for analysis by other investigators

Technological advances in the last decade have enabled both induction of precise changes in zebrafish nervous system form and function and the ability to better observe and characterize those changes. One of the most exciting developments in zebrafish neuroscience has been the coupling of light-sheet microscopy and optogenetic techniques to create images of exceptional resolution (Del Bene and Wyart, 2012; Kokel et al., 2013; Muto et al., 2013; Portugues et al., 2013, 2014), allowing researchers to stimulate behavior and observe effects at the cellular level in real time (Wyart et al., 2009). The genetic malleability of zebrafish has allowed delineation of function in selected brain regions or cells (Birely et al., 2005; Elbaz et al., 2012; Tabor et al., 2014; Zhao et al., 2009). As neurotoxicology moves forward, these techniques will improve our understanding of the environmental etiology of nervous system disorders.

## 7 Immunotoxicology

Immunotoxicology is the study of the immune response following exposure to natural and anthropogenic toxicants. It is well known that environmental stresses posed by environmental toxicants often increase an organism’s susceptibility to disease, and there is considerable evidence that this phenomenon is due to environmental toxicant effects on the immune response (Bols et al., 2001). A number of experiments have demonstrated the utility of fish models for immunotoxicity studies.

### 7.1 Overview of immunotoxicity studies using fish species

A variety of fish models have been used in studies of the effects of environmental toxicant exposure on the immune system. These studies have yielded valuable insights into the potential impact that exposure to environmental toxicants may have on human health. For example, studies using carp (*Cyprinus carpio*) and medaka showed how heavy metals and endocrine-disrupting chemicals alter the immune response and overall health (Betoulle et al., 2002; Dautremepuits et al., 2004; Ghanmi et al., 1993; Pham et al., 2012; Prophete et al., 2006; Sovenyi and Szakolczai, 1993; Steinhagen et al., 2004; Sun et al., 2011; Wang et al., 2011; Witeska and Kosciuk, 2003; Witeska and Wakulska, 2007). Other studies reported how environmental estrogens (Filby et al., 2007), hydroxylated fullerenes (Jovanovic et al., 2011a), and titanium dioxide (Jovanovic et al., 2011c) affect the ability of fathead minnows (*Pimephales promelas*) to mount an immune response; how herbicides, including atrazine, simazine, diuron, and isoproturon, suppress immune function in goldfish (*Carassius auratus*) (Fatima et al., 2007); and how arsenic administered to killifish disrupts the function of cystic fibrosis transmembrane conductance regulator, a known mediator of innate immunity (Bomberger et al., 2012; Shaw et al., 2010; Stanton et al., 2006). Other classes of chemicals found to alter the immune response through studies with zebrafish include endocrine-disrupting chemicals 17β-estradiol, 17α-ethynylestradiol, polychlorinated biphenyls, and bisphenols (Jin et al., 2010; Keiter et al., 2012; Li et al., 2013; Lyche et al., 2013; Tu et al., 2013; Xu et al., 2013); metals such as silver, gold, depleted uranium, and arsenic (Gagnaire et al., 2013, 2014; Hermann and Kim, 2005; Lage et al., 2006; Mattingly et al., 2009; Myrzakhanova et al., 2013; Nayak et al., 2007; Truong et al., 2013); and oxides such as titanium dioxide (Jovanovic et al., 2011b). These studies underscore the value of aquatic models in helping uncover the link between environmental exposures and immune dysfunction.

### 7.2 The zebrafish as a model system for immunotoxicology studies

Several features make zebrafish an attractive model for immunotoxicology studies, including high fecundity, embryonic and larval transparency, amenability to genetic manipulation (Kim and Tanguay, 2013), and a sequenced and annotated genome exhibiting large regions of conserved synteny with humans (Catchen et al., 2011). Regions of orthology and functional conservation between zebrafish and human genomes include genes related to blood cell development and immune function (Ellett and Lieschke, 2010; Savan and Sakai, 2006; Sullivan et al., 2007, 2009).

Zebrafish mount robust immune responses to infectious agents and rely upon their innate immune system in the first 4-6 weeks of development (Novoa and Figueras, 2012). During this window, no coordinated adaptive immune response is mounted, although some genes associated with adaptive immunity are expressed (Zapata et al., 2006). Therefore, the young zebrafish is an ideal model system in which to study the innate immune response without interference from an adaptive response. In fact, zebrafish deploy the phagocytic activity of macrophages and neutrophils to suppress infections in the same way humans do (Ginhoux and Jung, 2014; Henry et al., 2013). Transgenic zebrafish lines in which macrophages and neutrophils are tagged with fluorescent proteins (Ellett et al., 2011; Mathias et al., 2009; Renshaw et al., 2006) enable detailed real-time studies of phagocyte migration to sites of infection or inflammation. The transparency of the larva allows this migration to be directly observed (Henry et al., 2013; Mathias et al., 2009; Phennicie et al., 2010), enhancing our understanding of the phagocytic response. Not surprisingly, the zebrafish model has become increasingly popular for use in studies of innate immunity (Renshaw and Trede, 2012; Sullivan and Kim, 2008).

Zebrafish have been used to study a wide variety of bacterial, viral, and fungal infections (Brannon et al., 2009; Phennicie et al., 2010; Singer et al., 2010; Brothers and Wheeler, 2012; Chao et al., 2010; Gratacap et al., 2013). Inserting fluorescent transgenes into these pathogens enables real-time visualization of the infection process. In combination with transgenic zebrafish lines, phagocyte-pathogen interactions can be visualized dynamically in ways not possible in more conventional model organisms, including mice (Ramakrishnan, 2013).

As described in the preceding sections, targeted gene editing techniques have been developed for the zebrafish model system. These techniques have led to important insights into aspects of immune function such as blood cell development. Host-pathogen interactions have been studied in the presence or absence of a specific gene product using a variety of measurements, including whole-animal respiratory burst, pathogen burden, mortality, phagocyte population and migration, and cytokine profile assays (Bill et al., 2009; Boatman et al., 2013).

### 7.3 Using the zebrafish immunotoxicology model for chemical hazard prediction

Immunotoxicology studies using fish models in high-throughput systems have provided important insights into underlying mechanisms of toxicity. Fish studies allow convenient treatment with ecotoxicants, which can be added directly to the water of the developing embryos. Initial zebrafish Tox21 studies identified developmental morphology and neurotoxicity phenotypes associated with chemical exposure (Truong et al., 2014), as well as predictive linkages between neurotoxicity and morphology phenotypes measured at different developmental stages (Reif et al., 2015). These efforts have provided extraordinary insights and should be complemented with additional high-throughput methodologies. Multipronged approaches like those being employed by the Tox21 program leverage data from multiple organisms, including zebrafish, and cell lines to gain a multidimensional understanding of chemical toxicity (Kim and Tanguay, 2013). Continued development of these models should be fostered and expanded to include other complementary fish models.

## 8 Conclusions and recommendations

Fish and humans share similar developmental and physiological responses to chemical exposures. Unlike their rodent counterparts, small aquatic models such as zebrafish and medaka are amenable to high-throughput studies. Presentations at this workshop discussed the numerous advantages fish have over traditional mammalian models including small size, rapid development, high fecundity, and external development with similar physiology to that of traditional models. Given these advantages, use of these animals in toxicology has the potential to usher in a new era characterized by rapid *in vivo* screens that generate actionable data for use in regulatory settings aimed at improving human and environmental well-being. Significantly, these models can complement existing high-throughput *in vitro* cell- and receptor-based assays, which are excellent for providing mechanism of action insights but lack the ability to generate systems-level understanding of modes of action.

Workshop participants also identified ways in which these models could be strengthened and impediments to broader acceptance dispelled. Participants agreed that a better understanding of absorption, distribution, metabolism, and excretion in these species would improve the ability to extrapolate results to mammalian systems. Furthermore, as described above, use of these models is currently hampered by the lack of standardized protocols, an issue that must be addressed if these organisms are to move to the forefront of *in vivo* toxicology research. Development of standardized protocols will in turn facilitate validation. Validation should include both formal validation studies and an examination of the value of adding experiments using aquatic models to existing mammalian model-based testing. Workshop participants felt that, ultimately, regulatory acceptance would greatly promote broader utilization of aquatic models.

This workshop highlighted the importance of aquatic models, and in particular small fish models, in toxicology research and provided a roadmap for overcoming remaining obstacles to their successful incorporation into 21^st^ century toxicological research. Widespread support was indicated for convening follow-up workshops to include regulators and industry representatives to further explore the applicability and barriers to implementation of these models to regulatory toxicology testing. We anticipate that a follow-up meeting will take place in early 2017.

## Footnotes

1 http://oacu.od.nih.gov/ARAC/documents/Zebrafish.pdf

2 http://www.niehs.nih.gov/about/index.cfm

3 https://www.epa.gov/sites/production/files/2015-07/documents/850-1075.pdf

4 http://www.oecd.org/chemicalsafety/testing/36817242.pdf

5 https://zebrafish.org/home/guide.php

6 https://www.gwascentral.org

7 https://undiagnosed.hms.harvard.edu
